# Seasonal changes of buffalo colostrum: physicochemical parameters, fatty acids and cholesterol variation

**DOI:** 10.1186/1752-153X-7-40

**Published:** 2013-02-26

**Authors:** Aurelia Coroian, Silvio Erler, Cristian T Matea, Vioara Mireșan, Camelia Răducu, Constantin Bele, Cristian O Coroian

**Affiliations:** 1 Department of Food Science and Technology, University of Agricultural Sciences and Veterinary Medicine, Calea Mănăştur 3-5, 400372, Cluj-Napoca, Romania; 2 Department of Apiculture and Sericulture, University of Agricultural Sciences and Veterinary Medicine, Calea Mănăştur 3-5, 400372, Cluj-Napoca, Romania; 3 Department of Biochemistry, University of Agricultural Sciences and Veterinary Medicine, Calea Mănăştur 3-5, 400372, Cluj-Napoca, Romania; 4 Department of Anatomy and Animal Physiology, University of Agricultural Sciences and Veterinary Medicine, Calea Mănăştur 3-5, 400372, Cluj-Napoca, Romania

**Keywords:** Buffalo colostrum, Fatty acids, Cholesterol, GC, HPLC, Seasonal variation, Diet

## Abstract

**Background:**

Colostrum has many beneficial effects on newborns due to its main compounds (proteins, fats, lactose, essential fatty acids, amino acids) as well as protective antibodies that confer to the body. The buffaloes are the second important species for milk production in the world after cows. The importance of the species is also conferred by a longer longevity, high dry content of milk and a strong organic resistance when compared with cows. The purpose of this study was to investigate the changes of buffalo colostrum compounds such as fatty acids, cholesterol and physicochemical parameters during the first seven days *postpartum* and under the impact of the season, summer on pasture and winter on dry diet (hay based).

**Results:**

Fat from colostrum differs depending on the *postpartum* day showing mean values of 11.31-7.56% (summer season) and 11.22-7.51% (winter season). These values gradually decreased starting with first day *postpartum* until day seven. Dry substance and protein presented a similar evolution to fat reaching the lowest values at the end of the colostral period. Lactose, ash and pH showed a gradually increase reaching the maximum on day seven *postpartum*. The highest titres of fatty acids from colostrum are: butyric acid (C4:0), myristic acid (C14:0), palmitic acid (C16:0), oleic acid (C18:1) and the lowest values showed up in myristoleic acid (C14:1), cis-10-pentadecanoic acid (C15:1), pentadecylic acid (C15:0) and margaric acid (C17:0) for both seasons. Higher concentrations have been recorded for the summer season in general. Cholesterol concentration decreased from 12.93 and 12.68 mg/100 mL (summer and winter season) to 9.02 and 7.88 mg/100 mL in the end of the colostral period.

**Conclusions:**

Physicochemical compounds of buffalo colostrum were influenced by season and *postpartum* day of milking. Excepting lactose all other parameters gradually decreased during colostral period. Fatty acids and cholesterol showed the same evolution, presenting higher values for the summer season. Specific feeding in the summer season (on pasture) did lead in more concentrated colostrum in dry substance, fatty acids and cholesterol.

## Background

Colostrum is the first food for mammalian newborns during the first days of life. The composition of this liquid secreted and stored in the mammary gland in the first days after parturition is of great importance due to its physicochemical properties and its immunoprotective role in the early days of the calf [1]. Colostrogenesis, the transfer of immunoglobulines (Ig) from maternal circulation to mammary secretions, begins 3–4 weeks before parturition under endocrine control. Colostrum is critical for the survival of ruminant neonates since maternal antibodies are not transported across the placenta [2]. Colostrum is considered vital for normal growth and development of the calves [3] and it is also a protecting agent against various diseases [4-6].

Colostrum contains major nutrients (fatty acids, proteins, carbohydrates), vitamins (A, B6, B12, C etc.), minerals (Ca, Na, Mg, P, Cl, K etc.), immunological compounds (immunoglobulines – IgG, IgA and IgM) [5,7], hormones and enzymes [7,8]. Besides providing immune support, colostrum has remarkable musculoskeletal repair and growth capabilities. In addition, it seems that colostrum is the only natural source of four major growth factors namely transforming growth factors alpha (TGF-α) and beta (TGF-β), and insulin-like growth factors 1 (IGF-1) and 2 (IGF-2) [9].

The main parameters which are ordinary determined in almost every cow and/or buffalo farm are: fat, protein, lactose, total solids, ash and pH. The buffalo diet seems to have a major role in colostrum composition [10], but also the lactation, age, season, state of health, breed and genetic input of the parents [11-14].

Although colostrum is theoretically exclusively used for calves’ nutrition, the variation of its main compounds might provide useful information on the optimal length of colostral feeding of newborns. New insights on colostrum usage could aim the milking of extra-quantities that are not needed anymore for calves once the immunological effects are fulfilled. The usual colostral period for calves is 3–5 days, although Ig absorption ends in the first 36 h *postpartum*. Nevertheless, colostrum is a valuable source of other compounds for the newborn animal, such as proteins, fat, vitamins etc. Otherwise, we have to take in consideration colostrum supplements or colostrum replacers. Hence, bovine colostrum might be used as important source of fatty acids and other compounds in human diet.

The total lipids from buffalo milk are in a higher content [15] compared with cow milk but there are still missing studies on fatty acids (FA) from buffalo raw milk and even less from colostrum. Lipids contribute to sensorial and nutritional quality improvement of various products (mostly various types of cheese) and provide many benefits to human health, one of the most important being an anticarcinogenic agent [16].

Bovine milk fat contains approximately 400 different fatty acids, which makes it the most complex of all natural fats [17]. Fatty acids are an important nutrient [18], growth factor and involved in immune system fitness [19]. Fatty acids play an essential role in metabolism, providing the cell a concentrated source of energy and are implemented in the cell wall structure [20]. Some of relatively obscure fatty acids play a crucial role in growth and development [21].

Several fatty acids have beneficial effects on human health: butyric acid (C4:0) in cancer prevention [16,22]; medium-chain saturated and long-chain unsaturated fatty acids were all antiviral at different concentrations [23]; very long chain poly-unsaturated fatty acids of the n-3 family (e.g., eicosapentaenoic acids (C20:5n-3) and docosahexaenoic acids (C22:5n-3)) have an inhibiting effect on tumor development [24]; lauric acid (C12:0) may act as an antimicrobial agent [25]. Saturated fatty acids (SFA’s) in general should not exceed 10% of the total fat intake in human nutrition [26], because of negative aspects such as an increase in low-density lipoprotein cholesterol (LDL-C) and further the risk of coronary heart diseases [27]. The adults of ruminants (cows, buffaloes, sheep, goats and camels) can synthesize volatile fatty acids (VFA): acetic (C2:0), propionic (C3:0) and butyric (C4:0) acids in their rumen [28].

Diet, the effect of breed, evolution of lactation and variation between individuals are the major factors influencing fatty acid concentration in milk [29]. Seasonal variation of fatty acids content might provide useful information for the optimal harvesting period during the year in relation to the highest concentration of those fatty acids. Nutritional and metabolic effects of highly concentrated fatty acids in colostrum might generate large benefits in calves but even humans. The use of extra-quantity (the amount of colostrum secreted after the first 3–5 days) of fatty acid rich colostrum in human nutrition supplementation should not be neglected.

Cholesterol is an important compound in milk and acts as a critical component of cell membranes, the precursor of all steroid hormones, a precursor of vitamin D, and the limiting factor that brain cells need to make connections with one another called synapses, making it essential for learning and memory [30]. Its concentration is low (0.5% from the total lipids in human milk – [31,32] but higher in the first days of lactation – colostral period. Both, fatty acids and cholesterol concentrations in colostrum greatly vary depending on the species [33-36].

In the literature, there are studies on colostrum from cows, but there are no or poor studies on colostrum from buffaloes, which is in some countries as India, Pakistan and China the second most important species in milk production. One of the most interesting aspects concerning the nutrition of ruminant calfs in the first weeks of life is the non-functionality of specific ruminal digestion. Only the real stomach – *abomasum* – is active and therefore the direct intake of all nutritional compounds is demanded.

The purpose of this study was to determine the evolution of main physical and chemical compounds of buffalo colostrum as well as fatty acids (10 saturated, five mono-unsaturated fatty acids (MUFAs) and one poly-unsaturated fatty acid (PUFA)) and cholesterol within the first seven days *postpartum* depending on the season (pasture *vs*. dry diet). Although buffalo colostrum has been mainly studied for its immunological aspect given by immunoglobulin content [5,15], the nutritional and quality of the main nutritional compounds aspect cannot be neglected.

## Results and discussion

### Physicochemical characterization of buffalo colostrum according to seasonal variation

Comparing the main physicochemical parameters of buffalo colostrum from the summer and winter season a significantly increased titer in the summer season has been detected for most of them (Table 1, Additional file 1: Tables S1, S2). The overall trend during the first seven days is a rapid decrease of the main physicochemical colostrum parameters until day four; thereafter the slope is still decreasing, but negligible. This observation was similar for total solids, fat, and protein. In contrast is lactose, which constantly increased up to day four, followed by a slightly stagnation of the titer for the next three days. The same ascending trend for lactose and descending for protein and fat was reported by [15] for Egyptian buffalo colostrum in the first days after calving. In general, there seems to be no major difference in changes of the basic nutrition parameters between buffalos and cows. In Holstein cows the same general changes were measured [15]. In fact, the lactose concentration from colostrum still increases after the colostral period, reaching the highest values in regular milk [37]. It seems that energy given by lactose is more important in the first days of life and even for newborns than the fat and proteins, which is not surprising because carbohydrates show the highest respiratory quotient following proteins and fat. Analysis of variance for each compound between and within each season showed highly significant differences between days and seasons (Friedman ANOVA, *P* < 0.001, Additional file 1: Table S1 and Wilcoxon Matched Pairs Test, *P* < 0.0001, Additional file 1: Table S2). The lower values for the winter season might be explained by various reasons ranging from lower feeding level in high quality protein and energy, and rate of metabolic energy conversion. In contrast, pasture is well-balanced in the main nutritional compounds and has a higher conversion rate, conversely doubled by optimal climatic factors in the summer season.

**Table 1:** Evolution of the main chemical compounds of buffalo colostrum in summer (A) and winter (B)

	**Days *postpartum***						
**A**	**1**	**2**	**3**	**4**	**5**	**6**	**7**
**Fat (%)**	11.31±0.39	10.40±0.09	8.59±0.18	8.14±0.05	7.90±0.15	7.67±0.06	7.56±0.06
**Protein (%)**	8.73±0.15	7.27±0.06	7.01±0.05	5.83±0.02	5.62±0.02	5.31±0.02	5.24±0.03
**Lactose (%)**	3.73±0.02	3.84±0.03	3.93±0.02	4.96±0.02	4.99±0.02	5.09±0.01	5.11±0.01
**Total solids (%)**	25.31±0.02	22.53±0.05	20.41±0.02	19.83±0.05	19.40±0.02	19.17±0.02	19.01±0.03
**Ash (%)**	0.94±0.02	0.91±0.01	0.92±0.02	0.94±0.01	0.97±0.01	1.01±0.01	1.03±0.01
**pH**	6.01±0.01	6.09±0.01	6.36±0.02	6.40±0.01	6.44±0.01	6.50±0.01	6.63±0.03
**B**							
**Fat (%)**	11.22±0.38	10.35±0.05	8.44±0.05	8.11±0.03	7.86±0.13	7.66±0.07	7.51±0.03
**Protein (%)**	8.64±0.12	7.22±0.03	6.97±0.07	5.79±0.03	5.60±0.03	5.29±0.02	5.22±0.04
**Lactose (%)**	3.71±0.02	3.82±0.02	3.91±0.01	4.94±0.01	4.96±0.04	5.06±0.02	5.08±0.01
**Total solids (%)**	25.25±0.04	22.55±0.06	20.34±0.09	19.78±0.03	19.38±0.01	19.10±0.03	18.97±0.04
**Ash (%)**	0.91±0.02	0.88±0.02	0.90±0.02	0.91±0.02	0.96±0.01	0.97±0.03	1.01±0.02
**pH**	6.00±0.01	6.06±0.01	6.28±0.02	6.37±0.04	6.40±0.02	6.46±0.04	6.55±0.04

(All values are mean±SD and expressed in %; n = 4).

Consequently, the summer born calves have an advantage in a more concentrated colostrum feeding. Similar seasonal variation of basic parameters from colostrum occurred also in cows [38]. Compared to regular milk, colostrum has at least 40% more dry matter (in cows and buffaloes [15]; in buffaloes [39]; in sheep [40]). This rich milk could thus provide higher amounts of fats and proteins to their progenies and even human consumption.

Our results are in accordance with those reported by [6] which measured the same parameters to the same breed and exceed the data reported by [41] (which used buffalo breeds from Pakistan).

### Fatty acids and cholesterol variation in buffalo milk according to the season

Here, we determined sixteen fatty acids from buffalo colostrum, including one volatile fatty acid (butyric acid). The variation in fatty acid concentrations showed a significant descendent trend (Friedman ANOVA, *P* < 0.001) during the first seven days of lactation (Table 2, Additional file 1: Table S3). All colostrum fatty acids decreased by approximate 15-20% from the first to the seventh day, independent of the season, excepting palmitoleic acid (C16:1) which decreases rapidly until day four and further a slightly descend plateau was detected. The summer colostrum showed a much higher concentration for all studied fatty acids in relation to winter colostrum (Wilcoxon Matched Pairs Test, *P* < 0.0001, Additional file 1: Table S4). A comparison of saturated fatty acids (SFA), mono-unsaturated fatty acids (MUFA) and poly-unsaturated fatty acids (PUFA) for the two seasons evidenced a higher variation of saturated fatty acids compared with others. Seasonal variation also occurred in a comparative study on buffalo, cow, goat and sheep milk where the pasture season has shown a higher concentration of total poly-unsaturated (4-35%), trans- (16-35%) and conjugated linoleic acid contents (24-48%), while short fatty acids (< 14:0) showed an opposite effect [42]. It seems that naturally grown fresh grass during summer affects the bio-hydrogenation pathways [42]. In the colostral period the same physiological pathways seem to be present, reaching a higher concentrated fatty acid diet for calves. The metabolic needs in fatty acids of buffalo calves in the first days of life are unknown and a correlation with fatty acid titers in colostrum is impossible. However, it is the fact that in the first days of life the buffalo newborns only have one of the stomachs’ compartment active (*abomasum*) and direct feeding with the indispensable nutrients such as proteins (amino acids), fats (fatty acids and cholesterol), energy (lactose and lipids), vitamins and immunoglobulin is demanded.

**Table 2:** Daily fatty acid (μg/100 g) and cholesterol (mg/100 mL) variation of buffalo colostrum in summer (A) and winter (B)

**Acid name**	**Days *postpartum***						
**A**	**1**	**2**	**3**	**4**	**5**	**6**	**7**
**SFA**							
**Butyric acid (C4:0)**	481.38±0.9	471.58±1.1	461.59±1.2	442.85±1.0	417.01±2.2	381.35±1.3	359.53±1.1
**Caproic acid (C6:0)**	53.77±0.1	45.01±0.1	40.94±0.4	40.35±0.4	38.11±0.1	37.76±0.2	34.67±0.3
**Caprylic acid (C8:0)**	46.22±0.4	38.12±0.1	37.63±0.4	37.17±0.3	36.83±0.1	27.02±0.4	26.53±0.6
**Capric acid (C10:0)**	84.70±0.5	65.99±0.1	58.65±0.4	52.45±0.6	42.04±0.1	37.06±0.3	45.51±0.6
**Lauric acid (C12:0)**	90.32±0.6	81.65±0.5	70.76±0.7	76.12±0.4	63.09±0.3	60.18±0.2	50.88±0.6
**Myristic acid (C14:0)**	446.05±0.6	422.01±0.6	346.04±0.7	334.89±0.4	288.81±0.4	251.69±0.4	240.88±0.5
**Pentadecylic acid (C15:0)**	38.48±0.5	39.72±0.3	34.94±0.5	33.05±0.1	25.48±0.4	26.45±0.3	21.58±0.2
**Palmitic acid (C16:0)**	1557.74±0.7	1441.48±0.8	1344.67±0.5	1251.72±0.9	1150.89±0.8	1106.05±0.7	1110.20±0.2
**Margaric acid (C17:0)**	35.87±0.3	35.01±0.5	32.71±0.5	32.2±0.4	30.69±0.5	29.02±0.2	28.80±0.3
**Stearic acid (C18:0)**	333.90±0.4	332.37±0.7	315.09±0.6	310.79±0.5	297.10±0.4	285.25±1.0	278.17±1.4
**MUFA**							
**Myristoleic acid (C14:1)**	23.45±0.4	22.77±0.4	18.90±0.2	19.93±0.4	18.86±0.1	14.95±0.1	12.77±0.6
**Cis-10-pentadecanoic acid (C15:1)**	14.74±0.3	12.14±0.2	11.96±0.4	10.79±0.4	10.90±0.2	10.03±0.4	10.10±0.5
**Palmitoleic acid (C16:1)**	58.50±0.3	56.67±0.4	56.85±0.1	46.31±0.4	47.21±0.5	45.37±0.2	44.44±0.6
**Oleic acid (C18:1)**	987.89±0.8	931.55±0.8	911.89±0.7	865.47±0.8	856.64±0.5	831.52±0.7	797.26±0.6
**Elaidic acid (C18:1iso)**	44.91±0.4	43.72±0.4	42.77±0.6	41.64±0.4	40.96±0.8	38.44±0.3	37.83±0.2
**PUFA**							
**Linoleic acid (C18:2)**	78.31±0.8	75.62±0.4	72.19±0.5	70.93±0.3	62.95±0.9	60.93±0.7	56.90±0.5
**Cholesterol**	12.93±0.3	12.50±0.4	11.66±0.6	11.01±0.3	10.73±0.6	10.98±0.5	9.02±0.6
**B**							
**SFA**							
**Butyric acid (C4:0)**	480.49±0.4	469.98±0.5	455.24±0.3	441.67±0.8	415.30±0.3	371.30±0.9	353.18±1.5
**Caproic acid (C6:0)**	51.62±0.1	42.64±0.3	38.15±0.2	39.36±0.4	36.14±0.1	33.05±0.1	31.01±0.1
**Caprylic acid (C8:0)**	41.90±0.2	38.81±0.2	36.55±0.4	32.89±0.2	28.91±0.3	26.72±0.3	24.56±0.3
**Capric acid (C10:0)**	71.91±0.3	54.91±0.5	51.84±0.5	47.89±0.6	40.11±0.4	40.34±0.3	34.01±0.1
**Lauric acid (C12:0)**	80.68±0.6	75.95±0.5	70.55±0.6	66.09±0.7	57.87±0.2	56.04±0.6	45.99±0.2
**Myristic acid (C14:0)**	391.87±0.5	375.44±0.3	336.20±0.6	324.88±0.7	278.52±0.5	241.70±0.4	234.73±0.5
**Pentadecylic acid (C15:0)**	35.02±0.6	30.41±0.4	33.40±0.4	28.99±0.6	26.94±0.4	25.54±0.2	22.29±0.4
**Palmitic acid (C16:0)**	1423.09±1.6	1365.24±0.4	1311.18±0.6	1221.52±1.1	1046.80±1.2	987.56±0.7	1000.45±2.5
**Margaric acid (C17:0)**	34.50±0.5	33.90±0.7	31.81±0.3	31.12±0.5	29.81±0.7	27.90±0.5	27.87±0.2
**Stearic acid (C18:0)**	331.4±0.6	318.2±0.6	314.4±1.0	307.91±0.5	295.5±0.5	281.4±0.9	274.98±0.7
**MUFA**							
**Myristoleic acid (C14:1)**	20.74±0.3	19.29±0.6	16.81±0.2	15.02±0.7	14.89±0.3	13.88±0.5	10.85±0.2
**Cis-10-pentadecanoic acid (C15:1)**	13.34±0.3	12.48±0.4	11.70±0.2	10.51±0.4	10.55±0.4	10.02±0.4	9.38±0.4
**Palmitoleic acid (C16:1)**	56.81±0.4	56.33±0.5	55.07±0.6	45.70±0.4	41.09±0.8	41.37±0.8	40.53±0.3
**Oleic acid (C18:1)**	980.86±0.6	925.43±0.57	910.01±0.1	856.04±0.7	849.06±0.3	826.3±0.8	782.42±1.1
**Elaidic acid (C18:1iso)**	42.68±0.4	41.91±0.64	40.63±0.4	41.03±0.3	39.66±0.8	38.04±0.4	35.09±0.9
**PUFA**							
**Linoleic acid (C18:2)**	78.04±0.6	75.27±0.7	71.59±0.5	68.64±0.7	61.11±0.5	57.25±0.6	55.45±0.8
**Cholesterol**	12.68±0.4	11.23±0.6	11.49±0.3	10.88±0.7	9.06±0.7	7.88±0.5	8.03±0.5

(All values are mean±SD; n = 3).

Cholesterol levels in buffalo colostrum are also higher in the summer season (9.02-12.93 mg/100 mL) when compared with the winter season (7.88-12.68 mg/100 mL) (Table 2). Daily variation, starting with the first day after calving until day seven, showed a rapid decrease of cholesterol (Friedman ANOVA, *P* < 0.001) (Additional file 1: Table S3) down to 67% (average for summer and winter) in buffalo colostrum. Not only daily changes were observed but also the seasonal variation is quite high (*P* = 0.0001). In the summer season the concentration of cholesterol in colostrum decreased gradually and relatively constant down to day six and an abrupt decrease in day seven. In the winter season the downward trend is more emphasised, at least starting with day five. The overall decrease is even bigger than in the summer season (Wilcoxon Matched Pairs Test, *P* < 0.001) (Additional file 1: Table S4), with a maximum of 63% at day seven. Even after the colostral period the cholesterol concentration decreased constantly in mares [33]. The rapid decrease of cholesterol concentrations starting with day five might be an indicator of the colostral period length.

The colostral period has usually considered not covering more than three days, more important is immediate administration of colostrum to newborns and also the quality of colostrum [43]. Calves were usually fed by their mothers within the first seven days and just thereafter the milking period started. There is a second option to milk the rich milk from day four to day seven and use it for further purposes such as human alimentation. New approaches and understanding of cholesterol metabolism [44], and comparative studies on animal *vs*. industrial sources of fatty acids and cholesterol in human health [45], could clarify the importance of animals being sources of fatty acids and cholesterol in human nutrition.

## Conclusions

Some fatty acids and cholesterol from buffalo colostrum are significantly influenced by the season and were characterized by a high variability during the first seven days *postpartum*. All studied buffaloes showed the highest concentrations of fatty acids and cholesterol in the first five days of the colostral period, thereafter they gradually decreased, reaching normal parameters of buffalo milk at day seven. Higher concentrations of fatty acids and cholesterol during the summer season possibly have been influenced by pasture nutrition. Newborn calves born in summer could thus have more nutritional benefits and therefore a faster development comparing with winter ones. The buffaloes can be milked starting with day four as the calves already benefits by sufficient amount of colostrum and the surplus of this ‘rich’ milk till day seven could easily be used for human nutrition. The main physicochemical parameters also differed from summer to winter. Lactose was the only compound of buffalo colostrum which constantly increased during the first seven days of milking.

## Materials and methods

### Female individuals and colostrum sampling

Buffaloes selected for this study belonged to the Romanian buffalo breed, originated from the water buffalo. The experimental plots have been achieved from the experimental farm TNP Meşendorf, Braşov county, Romania. Each plot had 5 animals in successive lactations (third and fourth lactation). All animals have been fed *ad libitum* during the summer on pasture while in the winter season they were fed with hay and concentrates (cereals).

Colostrum samples were individually collected in the first seven days *postpartum*. The milk was collected in sterile containers and the milking was done manually. Samples for colostrum physicochemical analyses were immediately analyzed from fresh colostrum. Samples used for fatty acid and cholesterol analyses were kept at −20°C until analysis.

### Physicochemical analysis

The main physicochemical parameters have been determined with the milk analyzer LactoStar 3510 (Funke Gerber). Ash content was obtained following the method given in AOAC (2000) [46], while pH of raw milk was determined with a HI991300 pH meter from Hanna instruments at room temperature.

### Analytical methods

#### Fatty acids analysis

Total lipids were extracted using a modified Folch procedure [47] as described by Kraft et al., 2003 [48]. Briefly, 10 mL sample was used for extraction with 90 mL of a chloroform-methanol solution (2:1 v/v). Afterwards 30 mL of distilled water were added on top of the extract, into a separation funnel. After the separation of the two phases, the aqueous layer was discarded. The chloroformic fraction was further anhydrified using anhydrous sodium sulphate. The extracts were reduced nearly to dryness using a vacuum rotary evaporator at 35°C. Fatty acids were converted to methyl esters by reaction with boron trifluoride/methanol at 80°C for two hours in a closed Pyrex glass tube. Afterwards the sample was placed into a separation funnel were the esters were extracted using 10 mL hexane three times. The collected hexanic fractions were dried using anhydrous sodium sulphate, filtered, concentrated under a nitrogen stream and finally re-eluted in 1 mL hexane. Non-adecanoic acid (19:0) was used as internal standard for quantification purposes, which was added in each sample before the transesterification step. In order to identify the peaks, a FAME mix (Supelco® 37 Component FAME Mix) was injected prior to sample analysis.

**Figure 1:**
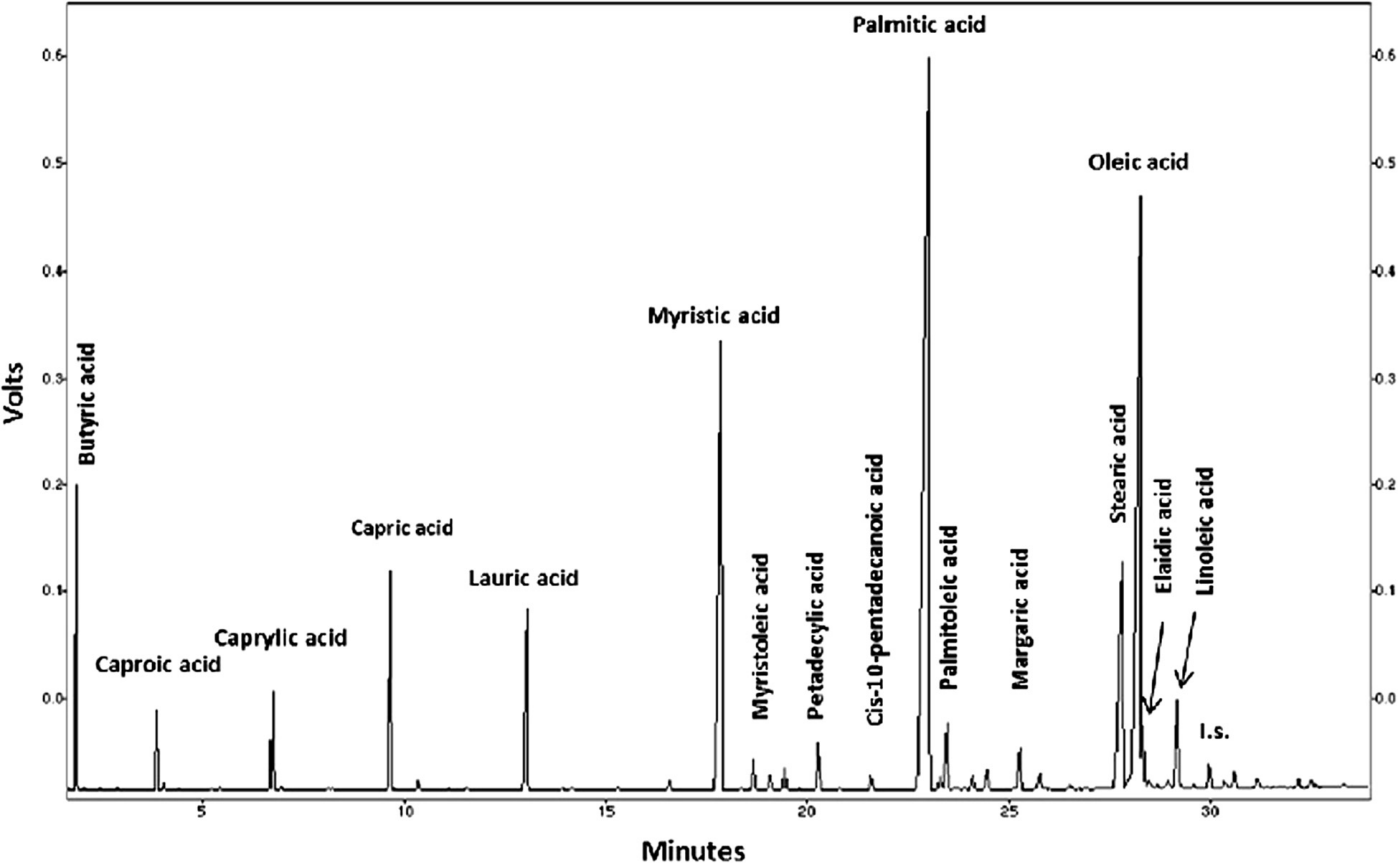
**Typical GC chromatogram of 16 fatty acids in buffalo colostrum.** C4:0 (Butyric acid), C6:0 (Caproic acid), C8:0 (Caprylic acid), C10:0 (Capric acid), C12:0 (Lauric acid), C14:0 (Myristic acid), C14:1 (Myristoleic acid), C15:0 (Pentadecylic acid), C15:1 (Cis-10-pentadecanoic acid), C16:0 (Palmitic acid), C16:1 (Palmitoleic acid), C17:0 (Margaric acid), C18:0 (Stearic acid), C18:1 (Oleic acid), C18:1 iso (Elaidic acid), C18:2 (Linoleic acid); I.s. – C19:0 (Non-adecylic acid – used as internal standard).

Fatty acids were analyzed by means of gas chromatography (GC) with flame ionization detection (FID) [49]. 1 μL sample was injected into the Shimadzu GC-17A series gas chromatograph, equipped with a 30 m polyethylene glycol coated column (Alltech AT-WAX, 0.25 mm I.D., 0.25 μm film thickness). Helium was used as carrier gas at a pressure of 147 kPa. For the oven temperature the following program was used: 70°C for 2 min then raised to 150°C at 10°C/min rate and held at 150°C for 3 min, then further raised up to 235°C at 4°C/min. The injector and detector temperatures were set at 260°C. The GC chromatogram for buffalo colostrum depicting fatty acids distribution is shown in Figure 1.

#### Cholesterol analysis

**Figure 2:**
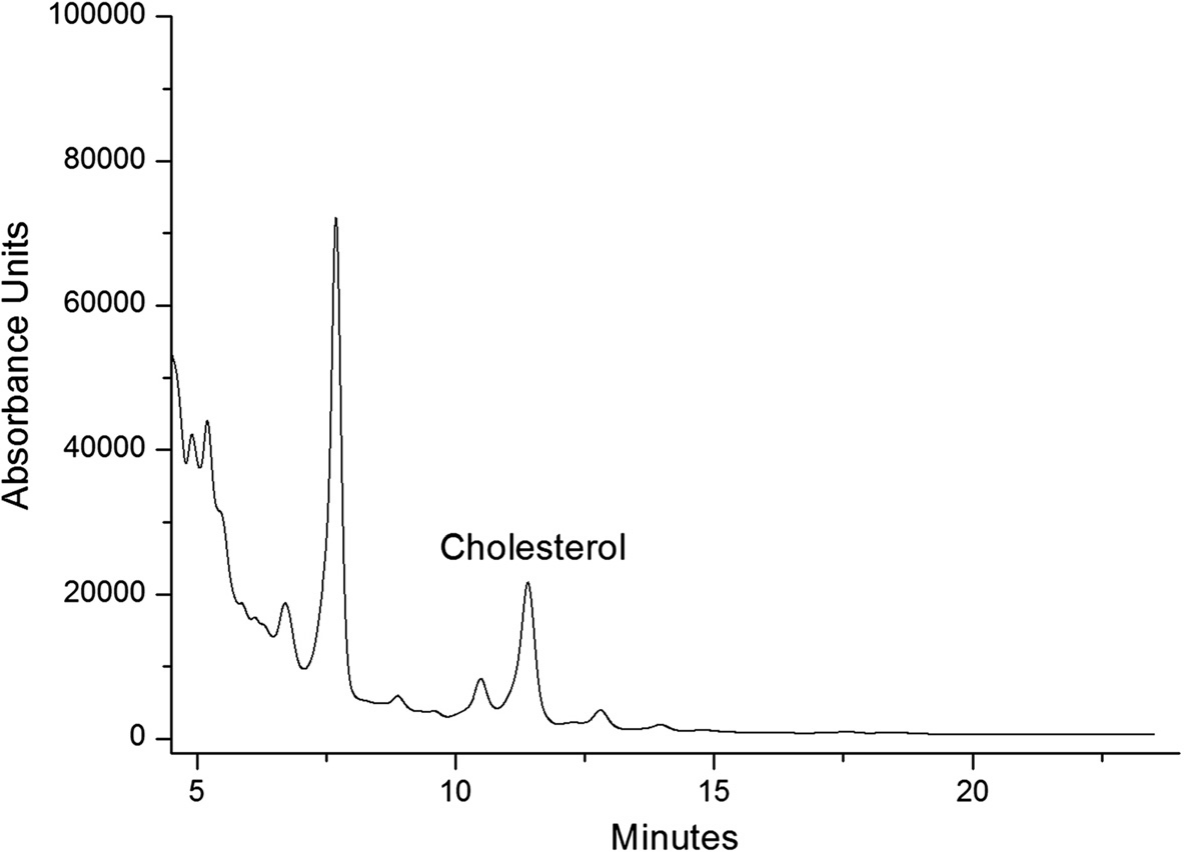
HPLC chromatogram from a buffalo colostrum sample detected at 210 nm.

The content of cholesterol was determined in accordance with a modified procedure described by Borkovcová et al., 2009 [50]. Briefly, a solution of 10 mol/L potassium hydroxide (9:1) was added to 10 mL of each sample and refluxed for 30 minutes. 5 mL deionized water and 10 mL n-hexane were added after cooling down to room temperature and the sample was intensively shaken for 20 minutes. The organic layer was retained and further washed with deionized water until neutral reaction and dried with sodium sulphate. The sample was reduced nearly to dryness using a vacuum rotary evaporator and re-dissolved in 1 mL acetonitrile-methanol (70:30 v/v). Quantification by HPLC was carried out using a Shimadzu VP Series liquid chromatograph system equipped with two delivery pumps and a UV–VIS detector at 210 nm. The chromatographic separation was carried out on an Alltima RP C-18 column (250 × 4.6 mm, 5 μm, Alltech Associates Inc.). Isocratic elution with a mobile phase of acetonitrile and isopropanol (70:30) mixture at a flow rate of 1 mL/min was used. Column temperature was set at 35°C and injection volume was 20 μL. An external calibration was performed prior to the analyses of the dairy products. The calibration curve was made with cholesterol concentrations ranging from 0.2 to 2 mg/mL (6 data points). The linear correlation between the cholesterol peak area and its concentration was satisfactory (*r*^*2*^ = 0.999). Recovery tests, detection and quantification values for this method are shown in Additional file 1: Table S5. A typical chromatogram for cholesterol found in buffalo colostrum is presented in Figure 2.

### Statistical analysis

Values were expressed as mean and standard deviation (SD) for all studied parameters. All statistical analyses were done using standard spreadsheet software and STATISTICA 8.0 (StatSoft, Tulsa, Oklahoma, USA). Level of statistical significance was defined as *P* < 0.05.

## Abbreviations

GC: Gas chromatograph;HPLC: High performance liquid chromatography;SFA: Saturated fatty acids;MUFA: Mono-unsaturated fatty acids;PUFA: Poly-unsaturated fatty acids

## Competing interests

The authors state that there are no competing interests.

## Supplementary Material

Additional file 1 **The following additional data are available with the online version of this paper.** Additional data file 1-**Table S1** is a table showing details on statistical analysis of seasonal variation of the main physicochemical parameters of buffalo colostrum during first seven days *postpartum*; Comparative analysis of the main physicochemical parameters of buffalo colostrum in summer and winter seasons; Statistical analysis of seasonal variation of some fatty acids and cholesterol from buffalo colostrum during first seven days *postpartum*; Comparative analysis of some fatty acids and cholesterol from buffalo colostrum in summer and winter seasons; Method validation for cholesterol quantification by HPLC-UV. Click here for file

## References

[B1] Singh A Ahuja SP Individual variation in the composition of colostrum and absorption of colostral antibodies by the precolostral buffalo calf J Dairy Sci 1993 76 1148 1156 10.3168/jds.S0022-0302(93)77443-3 8486842

[B2] Barington GM McFadden TB Huyler MT Besser TE Regulation of colostrogenesis in cattle Livest Prod Sci 2001 70 95 104 10.1016/S0301-6226(01)00201-9

[B3] Blum JW Hammon H Colostrum effects on the gastrointestinal tract, and on nutritional, endocrine and metabolic parameters in neonatal calves Livest Prod Sci 2000 66 151 159 10.1016/S0301-6226(00)00222-0

[B4] Anantakrishan CP Bhale Rao VR Paul TM Rangaswamy MC The component fatty acids of buffalo colostrum fat J Biol Chem 1946 166 31 33 20273670

[B5] Dang AK Kapila S Purohit M Singh C Changes in colostrum of Murrah buffaloes after calving Trop Anim Health Prod 2009 41 1213 1217 10.1007/s11250-008-9302-7 19105042

[B6] Velea C Zanc C Creşterea şi exploatarea bubalinelor 2011 Bucureşti: Editura Texte

[B7] Elfstrand L Lindmark-Månsson H Paulsson M Nyberg L Åkesson B Immunoglobulins, growth factors and growth hormone in bovine colostrum and the effects of processing Int Dairy J 2002 12 879 887 10.1016/S0958-6946(02)00089-4

[B8] Shahani KM Harper WJ Jensen RG Parry RM Jr Zittle CA Enzymes in bovine milk: a review J Dairy Sci 1973 56 531 543 10.3168/jds.S0022-0302(73)85216-6 4351390

[B9] Uruakpa FO Ismond MAH Akobundu ENT Colostrum and its benefits: a review Nutr Res 2002 22 755 767 10.1016/S0271-5317(02)00373-1

[B10] Panigrahi B Pandey HN Pattanaik AK Effect of pre-partum feeding of crossbred cows on growth performance, metabolic profile and immune status of calves Asian-Aust J Anim Sci 2005 18 661 666

[B11] Arumughan C Narayanan KM Influence of stage of lactation on the triacylglycerol composition of buffalo milk fat Lipids 1981 3 155 164 7219086 10.1007/BF02535433

[B12] Maunsell FP Morin DE Constable PD Hurley WL McCoy GC Kakoma I Isaacson RE Effects of mastitis on the volume and composition of colostrum produced by Holstein cows J Dairy Sci 1998 5 1291 1299 9621231 10.3168/jds.S0022-0302(98)75691-7

[B13] Farmer C Quesnel H Nutritional, hormonal, and environmental effects on colostrum in sows J Anim Sci 2009 87 56 65 18791139 10.2527/jas.2008-1203

[B14] Zarcula S Cernescu H Mircu C Tulcan C Morvay A Baul S Popovici D Influence of breed, parity and food intake on chemical composition of first colostrum in cow Anim Sci Biotechnol 2010 43 154 157

[B15] El-Fattah AMA Abd Rabo FHR El-Dieb SM El-Kashef HA Changes in composition of colostrum of Egyptian buffaloes and Holstein cows BMC Vet Res 2012 8 19 10.1186/1746-6148-8-19 22390895 PMC3344693

[B16] Parodi PW Cows' milk fat components as potential anticarcinogenic agents J Nutr 1997 6 1055 1060 9187617 10.1093/jn/127.6.1055

[B17] Månsson HL Fatty acids in bovine milk fat 2008 Nutr Res: Food 10.3402/fnr.v52i0.1821 PMC2596709 19109654

[B18] Makrides M Neumann M Simmer K Pater J Gibson R Are long-chain polyunsaturated fatty acids essential nutrients in infancy? Lancet 1995 345 1463 1468 10.1016/S0140-6736(95)91035-2 7769900

[B19] Yaqoob P Fatty acids as gatekeepers of immune cell regulation Trends Immunol 2003 24 639 645 10.1016/j.it.2003.10.002 14644137

[B20] Developmental role for fatty acids in Eukaryotes PLoS Biol 2004 2 293 10.1371/journal.pbio.0020293

[B21] Kniazeva M Crawford QT Seiber M Wang CY Han M Monomethyl branched-chain fatty acids play an essential role in *Caenorhabditis elegans* development PLoS Biol 2004 2 e257 10.1371/journal.pbio.0020257 15340492 PMC514883

[B22] German JB Butyric acid: a role in cancer prevention Nutr Bull 1999 24 203 209 10.1111/j.1467-3010.1999.tb00910.x

[B23] Thormar H Isaacs CE Brown HR Barshatzky MR Pessolano T Inactivation of enveloped viruses and killing of cells by fatty acids and monoglycerides Antimicrob Agents Chemother 1987 31 27 31 10.1128/AAC.31.1.27 3032090 PMC174645

[B24] De Vries CE van Noorden CJ Effects of dietary fatty acid composition on tumor growth and metastasis Anticancer Res 1992 12 1513 1522 1444214

[B25] Kabara JJ Swieczkowski DM Conley AJ Truant JP Fatty acids and derivatives as antimicrobial agents Antimicrob Agents Chemother 1972 2 23 28 10.1128/AAC.2.1.23 4670656 PMC444260

[B26] FAO Fats and fatty acids in human nutrition 2010 Geneva: Report of an expert consultation 10 14 November 2010

[B27] Baum SJ Kris-Etherton PM Willett WC Lichtenstein AH Rudel LL Maki KC Whelan J Ramsden CE Block RC Fatty acids in cardiovascular health and disease: a comprehensive update J Clin Lipidol 2012 6 216 234 10.1016/j.jacl.2012.04.077 22658146 PMC9398216

[B28] Dijkstra J Boer H Van Bruchem J Bruining M Tamminga S Absorption of volatile fatty acids from the rumen of lactating dairy cows as influenced by volatile fatty acid concentration, pH and rumen liquid volume Br J Nutr 1993 69 385 396 10.1079/BJN19930041 8489996

[B29] Kelsey JA Corl BA Collier RJ Bauman DE The effect of breed, parity, and stage of lactation on conjugated linoleic acid (CLA) in milk fat from dairy cows J Dairy Sci 2003 86 2588 2597 10.3168/jds.S0022-0302(03)73854-5 12939083

[B30] Schreurs BG The effects of cholesterol on learning and memory Neurosci Biobehav Rev 2010 34 1366 1379 10.1016/j.neubiorev.2010.04.010 20470821 PMC2900496

[B31] Jensen RG Ferris AM Lammi-Keefe CJ Henderson RA Lipids of bovine and human milks: a comparison J Dairy Sci 1990 73 223 240 10.3168/jds.S0022-0302(90)78666-3 2184172

[B32] Jensen RG Lipids in human milk Lipids 1999 34 1243 1271 10.1007/s11745-999-0477-2 10652985

[B33] Blasi F Montesano D De Angelis M Maurizi A Ventura F Cossignani L Simonetti MS Damiani P Results of stereospecific analysis of triacylglycerol fraction from donkey, cow, ewe, goat and buffalo milk J Food Comp Anal 2008 21 1 7 10.1016/j.jfca.2007.06.005

[B34] Pikul J Wójtowski J Fat and cholesterol content and fatty acid composition of mares' colostrums and milk during five lactation months Livest Sci 2008 113 285 290 10.1016/j.livsci.2007.06.005

[B35] Górová R Pavlíková E Blaško J Meľuchová B Kubinec R Margetín M Soják L Temporal variations in fatty acid composition of individual ewes during first colostrum day Small Ruminant Res 2011 95 104 112 10.1016/j.smallrumres.2010.09.005

[B36] Marounek M Pavlata L Mišurová L Volek Z Dvořák R Changes in the composition of goat colostrum and milk fatty acids during the first month of lactation Czech J Anim Sci 2012 57 28 33

[B37] Ontsouka CE Bruckmaier RM Blum JW Fractionized milk composition during removal of colostrum and mature milk J Dairy Sci 2003 86 2005 2011 10.3168/jds.S0022-0302(03)73789-8 12836936

[B38] Klimeš J Jagoš P Bouda J Gajdůšek S Basic qualitative parameters of cow colostrum and their dependence on season and postpartum time Acta Vet Brno 1986 55 23 39 10.2754/avb198655010023

[B39] Arain HH Khaskheli M Arain MA Soomro AH Nizamani AH Heat stability and quality characteristics of postpartum buffalo milk Pak J Nutr 2008 7 303 307 10.3923/pjn.2008.303.307

[B40] Or-Rashid MM Fisher R Karrow N Alzahal O McBride BW Fatty acid profile of colostrum and milk of ewes supplemented with fish meal and the subsequent plasma fatty acid status of their lambs J Anim Sci 2010 88 2092 2102 10.2527/jas.2009-1895 20154155

[B41] Anwar G Hanjra SH Khan BB Aborad Z A comparative study of cholostrum of buffalo and cow Pakistan J Agr Sci 1976 13 209 212

[B42] Talpur FN Bhanger MI Khooharo AA Memon GZ Seasonal variation in fatty acid composition of milk from ruminants reared under the traditional feeding system of Sindh, Pakistan Livest Sci 2008 118 166 172 10.1016/j.livsci.2008.04.008

[B43] Hammon HM Blum JW Free amino acids in plasma of neonatal calves are influenced by feeding colostrum for different durations or by feeding only milk replacer J Anim Physiol Anim Nutr 1999 82 193 204 10.1046/j.1439-0396.1999.00229.x 9482773

[B44] Van der Wulp MYM Verkade HJ Groen AK Regulation of cholesterol homeostasis Mol Cell Endocrinol 2012 10.1016/j.mce.2012.06.007 22721653

[B45] Brouwer IA Wanders AJ Katan MB Effect of animal and industrial trans fatty acids on HDL and LDL cholesterol levels in humans – a quantitative review PLoS ONE 2010 5 e9434 10.1371/journal.pone.0009434 20209147 PMC2830458

[B46] AOAC Association of Official Analytical Chemists 2000 Washington DC: Official Methods of Analysis, 17th Edition

[B47] Folch J Lees M Sloane-Stanley GH A simple method for the isolation and purification of total lipides from animal tissues J Biol Chem 1957 226 497 509 13428781

[B48] Kraft J Collomb M Möckel P Sieber R Jahreis G Differences in CLA isomer distribution of cow's milk lipids Lipids 2003 38 657 664 10.1007/s11745-003-1111-z 12934676

[B49] Meier S Mjøs SA Joensen H Grahl-Nielsen O Validation of a one-step extraction/methylation method for determination of fatty acids and cholesterol in marine tissues J Chromatogr A 2006 1104 291 298 10.1016/j.chroma.2005.11.045 16343517

[B50] Borkovcová I Janoušková E Dračková M Janštová B Vorlová L Determination of sterols in dairy products and vegetable fats by HPLC and GC methods Czech J Food Sci 2009 27 S217 219

