# Supplementary material for: Seasonal changes of buffalo colostrum: physicochemical parameters, fatty acids and cholesterol variation
Source: Chem Cent J. 2013 Feb 26;7:40. doi: 10.1186/1752-153X-7-40 (PMC3604957; doi:10.1186/1752-153X-7-40)
Supplement: Additional file 1 — The following additional data are available with the online version of this paper. Additional data file 1-Table S1 is a table showing details on statistical analysis of seasonal variation of the main physicochemical parameters of buffalo colostrum during first seven days postpartum; Comparative analysis of the main physicochemical parameters of buffalo colostrum in summer and winter seasons; Statistical analysis of seasonal variation of some fatty acids and cholesterol from buffalo colostrum during first seven days postpartum; Comparative analysis of some fatty acids and cholesterol from buffalo colostrum in summer and winter seasons; Method validation for cholesterol quantification by HPLC-UV. [file 1752-153X-7-40-S1.pdf]

Supplementary material

Table S1 - Statistical analysis of seasonal variation of the main physicochemical parameters of buffalo colostrum during first seven days *postpartum* (Friedman ANOVA – differences between days)

**Physicochemical parameters**	**change (day)**	**Overall** (N = 10, *df* = 6)		**Summer** (N = 5, *df* = 6)		**Winter** (N = 5, *df* = 6)	
		χ^2	***P***	χ^2	***P***	χ^2	***P***
**Fat**	4 ↓	59.61	< 0.0001	29.66	< 0.0001	30	< 0.0001
**Protein**	4 ↓	60	< 0.0001	30	< 0.0001	30	< 0.0001
**Lactose**	4 ↑	58.41	< 0.0001	29.31	< 0.0001	29.14	< 0.0001
**Total solids**	4 ↓	60	< 0.0001	30	< 0.0001	30	< 0.0001
**Ash**	2 ↑	50.80	< 0.0001	26.77	0.0002	25.18	0.0003
**pH**	3 ↑	58.37	< 0.0001	29.91	< 0.0001	2862	< 0.0001

Tabel S2 - Comparative analysis of the main physicochemical parameters of buffalo colostrum in summer and winter seasons (Wilcoxon Matched Pairs Test – summer vs. winter)

**Physicochemical parameters**	**Higher in**	***Z***	***P***
**Fat**	summer	4.169	< 0.0001
**Protein**	summer	5.119	< 0.0001
**Lactose**	summer	4.324	< 0.0001
**Total solids**	summer	4.078	< 0.0001
**Ash**	summer	5.086	< 0.0001
**pH**	summer	4.342	< 0.0001

Table S3 - Statistical analysis of seasonal variation of some fatty acids and cholesterol from buffalo colostrum during first seven days *postpartum* (Friedman ANOVA – differences between days)

**Acid**	**change**	**Overall** (N = 10, *df* = 6)		**Summer** (N = 5, *df* = 6)		**Winter** (N = 5, *df* = 6)	
		χ^2	***P***	χ^2	***P***	χ^2	***P***
**SFA**							
**Butyric acid (C4:0)**	↓	60	< 0.0001	30	< 0.0001	30	< 0.0001
**Caproic acid (C6:0)**	↓	58.93	< 0.0001	30	< 0.0001	30	< 0.0001
**Caprylic acid (C8:0)**	↓	58.16	< 0.0001	30	< 0.0001	28.46	< 0.0001
**Capric acid (C10:0)**	↓	56.1	< 0.0001	30	< 0.0001	29.49	< 0.0001
**Lauric acid (C12:0)**	↓	57.86	< 0.0001	30	< 0.0001	30	< 0.0001
**Myristic acid (C14:0)**	↓	60	< 0.0001	30	< 0.0001	30	< 0.0001
**Pentadecylic acid (C15:0)**	↓	55.82	< 0.0001	29.91	< 0.0001	30	< 0.0001
**Palmitic acid (C16:0)**	↓	60	< 0.0001	30	< 0.0001	30	< 0.0001
**Margaric acid (C17:0)**	↓	57.6	< 0.0001	28.97	< 0.0001	29.14	< 0.0001
**Stearic acid (C18:0)**	↓	60	< 0.0001	30	< 0.0001	30	< 0.0001
**MUFA**							
**Myristoleic acid (C14:1)**	↓	55.46	< 0.0001	29.23	< 0.0001	28.89	< 0.0001
**Cis-10-pentadecanoic acid (C15:1)**	↓	53.01	< 0.0001	27.09	< 0.0001	26.91	< 0.0001
**Palmitoleic acid (C16:1)**	↓ day 4	54.21	< 0.0001	29.49	< 0.0001	27.77	0.0001
**Oleic acid (C18:1)**	↓	60	< 0.0001	30	< 0.0001	30	< 0.0001
**Elaidic acid (C18:1iso)**	↓	58.59	< 0.0001	29.66	< 0.0001	29.49	< 0.0001
**PUFA**							
**Linoleic acid (C18:2)**	↓	60	< 0.0001	30	< 0.0001	30	< 0.0001
**Cholesterol**	↓	52.93	< 0.0001	29.66	< 0.0001	26.66	0.0002

Tabel S4 - Comparative analysis of some fatty acids and cholesterol from buffalo colostrum in summer and winter seasons (Wilcoxon Matched Pairs Test – summer vs. winter)

**Acid**	**Higher in**	***Z***	***P***
**SFA**			
**Butyric acid (C4:0)**	summer	4.783	< 0.0001
**Caproic acid (C6:0)**	summer	5.143	< 0.0001
**Caprylic acid (C8:0)**	summer	4.39	< 0.0001
**Capric acid (C10:0)**	summer	4.472	< 0.0001
**Lauric acid (C12:0)**	summer	5.127	< 0.0001
**Myristic acid (C14:0)**	summer	5.159	< 0.0001
**Pentadecylic acid (C15:0)**	summer	3.522	< 0.0001
**Palmitic acid (C16:0)**	summer	5.159	< 0.0001
**Margaric acid (C17:0)**	summer	5.127	< 0.0001
**Stearic acid (C18:0)**	summer	5.061	< 0.0001
**MUFA**			
**Myristoleic acid (C14:1)**	summer	5.159	< 0.0001
**Cis-10-pentadecanoic acid (C15:1)**	summer	2.488	0.0129
**Palmitoleic acid (C16:1)**	summer	5.012	< 0.0001
**Oleic acid (C18:1)**	summer	5.159	< 0.0001
**Elaidic acid (C18:1iso)**	summer	4.897	< 0.0001
**PUFA**			
**Linoleic acid (C18:2)**	summer	4.422	< 0.0001
**Cholesterol**	summer	4.832	< 0.0001

Table S5 - Method validation for cholesterol quantification by HPLC-UV (n = 3)

**Sample**	**mg/L**	**Recovery (%)**
**Intra-day**	40	98±5
	80	93±4
**Interday**	40	96±4
	80	92±4
**LOD (mg/L)**	1.97	
**LOQ (mg/L)**	6.56	

LOD: limit of detection calculated with a signal to noise ratio 3 to 1
LOQ: limit of quantification calculated with a signal to noise ratio of 10 to 1
